# Machine Learning-Driven Structural Optimization of a Bistable RF MEMS Switch for Enhanced RF Performance

**DOI:** 10.3390/mi16060680

**Published:** 2025-06-04

**Authors:** J. Joslin Percy, S. Kanthamani, S. Mohamed Mansoor Roomi

**Affiliations:** Department of ECE, Thiagarajar College of Engineering, Madurai 625015, India; skmece@tce.edu (S.K.); smmroomi@tce.edu (S.M.M.R.)

**Keywords:** RF MEMS switch, bistable lateral switch, I-clamp design, machine learning, XGBoost, structural optimization, activation functions

## Abstract

In the rapidly advancing digital era, the demand for miniaturized and high-performance electronic devices is increasing, particularly in applications such as wireless communication, unmanned aerial vehicles, and healthcare devices. Radio-frequency microelectromechanical systems (RF MEMS), particularly RF MEMS switches, play a crucial role in enhancing RF performance by providing low-loss, high-isolation switching and precise signal path control in reconfigurable RF front-end systems. Among different configurations, electrothermally actuated bistable lateral RF MEMS switches are preferred for their energy efficiency, requiring power only during transitions. This paper presents a novel approach to improve the RF performance of such a switch through structural modifications and machine learning (ML)-driven optimization. To enable efficient high-frequency operation, the H-clamp structure was re-engineered into various lateral configurations, among which the I-clamp exhibited superior RF characteristics. The proposed I-clamp switch was optimized using an eXtreme Gradient Boost (XGBoost) ML model to predict optimal design parameters while significantly reducing the computational overhead of conventional EM simulations. Activation functions were employed within the ML model to improve the accuracy of predicting optimal design parameters by capturing complex nonlinear relationships. The proposed methodology reduced design time by 87.7%, with the optimized I-clamp switch achieving −0.8 dB insertion loss and −70 dB isolation at 10 GHz.

## 1. Introduction

The digital era of rapid technological progress and communication advancements undeniably demands miniaturized electronic devices with enhanced performance [1,2]. This demand is prominently evident in applications such as wireless communication systems [3], unmanned aerial vehicles [4], and healthcare devices [5], where compact, high-performance solutions are indispensable. Microelectromechanical systems (MEMS) have emerged as a transformative technology in modern electronics, revolutionizing various industries with their miniature size, low power consumption, and exceptional performance characteristics [6,7]. The integration of MEMS devices such as switches [8], variable capacitors [9], resonators [10], filters [11], phase shifters [12], antennas [13], tunable inductors [14], voltage-controlled oscillators (VCOs) [15], and power amplifiers [16], especially within radio-frequency (RF) systems, has significantly advanced wireless communication technologies, offering enhanced functionality and efficiency.

RF MEMS switches transformed wireless communication by offering dynamic and efficient RF path selection, essential for adaptable circuits, spectrum management, and advanced signal processing [17]. Their rapid switching capability enhances performance in cellular networks, satellite communications, radars, and IoT devices, marking a significant leap in the wireless technology evolution [18,19,20]. RF MEMS switches play a vital role in advanced systems such as reconfigurable systems by enabling real-time switching between multiple RF paths, facilitating dynamic reconfiguration of antennas, filters, and matching networks with minimal signal loss and high isolation [13,21].

Recent advancements in RF MEMS switches have led to the development of various configurations categorized by actuation mechanisms (electrothermal, electrostatic, magnetostatic, electromagnetic, piezoelectric), structural designs (cantilever and fixed–fixed beams), and circuit topologies (series and shunt), operating through capacitive or ohmic contact interfaces with either vertical or lateral mechanical deflection [22,23]. In [24,25,26,27,28,29,30,31,32,33], a significant breakthrough was the development of electrothermally actuated bistable lateral RF MEMS switches. Such bistable switches combine the efficiency of electrothermal actuation with the stability of a bistable mechanism, resulting in a device that can maintain its state without the need for a constant power supply [29]. The lateral deflection of a bistable switch allows for a compact design and reduces the overall footprint on chips, making it highly suitable for integrated circuits where space efficiency is a paramount goal [32]. The bistability characteristics greatly enhance power efficiency, as they eliminate the need for continuous input power to maintain the switch state, thereby saving energy and prolonging battery life in portable devices [25]. The reliability of such a bistable switch is significantly improved due to the reduced wear and tear from constant switching, offering a longer operational lifespan.

In our previous work [34], the development of a bistable RF MEMS switch demonstrating robust performance at lower frequencies was reported. However, the challenge now lies in optimizing these switches for higher-frequency applications, where the requirements for insertion loss and isolation become more stringent. Addressing these challenges necessitates innovative approaches to design and optimize the switch, aiming to enhance operational efficacy while meeting the rigorous demands of high-frequency applications. To overcome these limitations, this paper adopts a novel approach by implementing structural modifications tailored to enhance the RF performance of bistable RF MEMS switches. The conventional H-clamp is replaced with various lateral clamp designs, among which the I-clamp exhibits improved RF characteristics, including lower insertion loss and higher isolation. However, further optimization of the bistable switch structure is essential to achieve enhanced performance across a broader frequency range. The optimization of such complex RF MEMS switches is time-consuming using 3D electromagnetic (EM) simulations [35]. In the past decade, machine learning (ML) algorithms were utilized to expedite the optimization process and achieve superior RF performance [36,37,38,39,40]. Recently, a pulse-driven MEMS gas sensor integrated with machine learning models such as linear discriminant analysis (LDA), k-nearest neighbors (KNN), and support vector machine (SVM) achieved high accuracy in selective gas identification, offering a compact, low-power solution for IoT applications [41]. Similarly, a multiband THz MIMO antenna employed gradient boosting regression for isolation prediction, attaining an over 98% accuracy and validating performance through both CST and ADS simulations [42]. In [43], Bayesian regularization-based ML models were used to validate optimal process parameters in RF MEMS glass via drilling, enhancing quality characteristics through metaheuristic algorithms, such as genetic algorithm (GA) and particle swarm optimization (PSO). A cognitive computing framework was proposed to predict the flow status of a flexible rectifier using machine learning models such as Multilayer Perceptron (MLP) and CatBoost, demonstrating superior fluidic prediction accuracy compared to traditional methods [44]. A multimodal strain-sensing system was developed for tensegrity structures, where deep learning-assisted shape recognition using long short-term memory (LSTM) networks enabled real-time structural deformation tracking [45]. These studies illustrate the increasing utilization of ML techniques to enhance functional predictability and structural adaptability in microstructured systems. Based on these concepts, the present work explores ML-driven optimization specifically tailored for bistable RF MEMS switches, focusing on RF performance enhancement through structural modifications and regression-based modeling. Through iterative learning and data-driven modeling, the ML approach facilitates the exploration of a vast design space, leading to the identification of optimal switch configurations that maximize RF efficiency. To capture complex relationships and patterns in the dataset, activation functions play a critical role in shaping the learning dynamics of the ML model [46,47,48]. A distinguishing aspect of our work lies in the comprehensive comparative analysis of activation functions within the context of ML-based optimization for RF MEMS switch applications. The objective of this work was to present a systematic framework for improving the RF performance of bistable RF MEMS switches through a synergistic combination of structural modification and ML-based optimization.

The major contributions of this paper include the following:

- Proposal of a novel I-clamp bistable RF MEMS switch to obtain better performance at higher frequencies;
- Development of an ML-based optimization model for the I-clamp bistable RF MEMS switch structure to minimize RF losses and improve isolation, enabling faster design convergence compared to traditional EM simulation-driven methods;
- Implementation of activation functions to model nonlinear relationships between structural parameters and RF performance in ML-based optimization of the proposed RF MEMS switch.

This paper is organized as follows. Section 2 describes the electrothermally actuated bistable lateral RF MEMS switch structure, its operation, and the need for structural modification. Section 3 deals with direct and inverse modeling of ML to predict the optimal geometrical parameters of a bistable RF MEMS switch. Section 4 presents the results and the discussion of the proposed ML model, followed by the conclusions in Section 5.

## 2. Modeled Device—Electrothermally Actuated Bistable RF MEMS Switch

### 2.1. Structural Description

**Figure 1:**
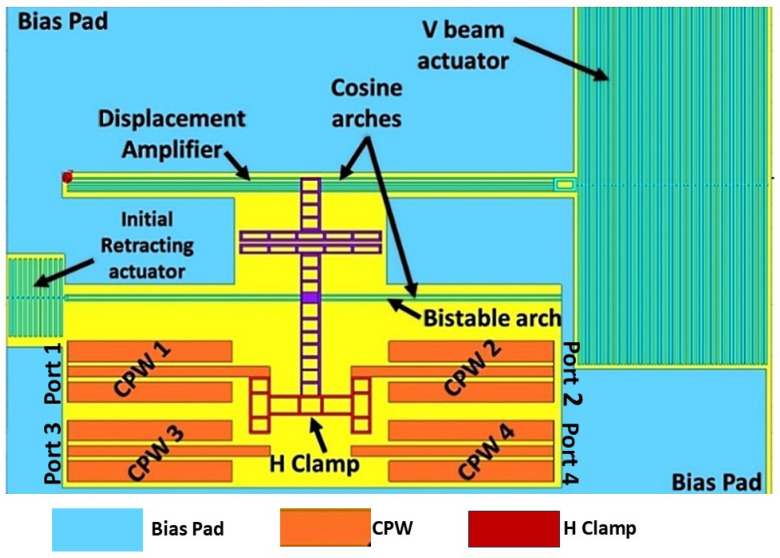
Structure of an electrothermally actuated H-Clamp bistable RF MEMS switch.

Figure 1 illustrates the structure of an electrothermally actuated bistable RF MEMS switch on a silicon substrate [29,34]. This structure comprises three main elements: two curved beams labeled as cosine arches, a retracting actuator, and a V-beam actuator. The upper cosine arch amplifies displacement, while the lower one is utilized as the bistable component. Coplanar waveguide (CPW) lines which perform RF signal transmission are strategically placed at the lower section of the device. They are seamlessly integrated with an intricate ladder structure of the arch and along with an extended structure called the H-clamp to establish lateral connections across the CPW lines on both sides.

### 2.2. Working Mechanism

The operation of a bistable RF MEMS switch begins in its initial OFF state, which is STABLE STATE 1. The transition mechanism from STABLE STATE 1 to STABLE STATE 2 depends on the application of pulse voltage across specific terminals. This triggers electrothermal actuation, simultaneously activating both the initially retracting actuator and the V-beam actuators. As a result, the bistable arch undergoes a lateral movement, transitioning into its STABLE STATE 2 [29].

**Figure 2:**
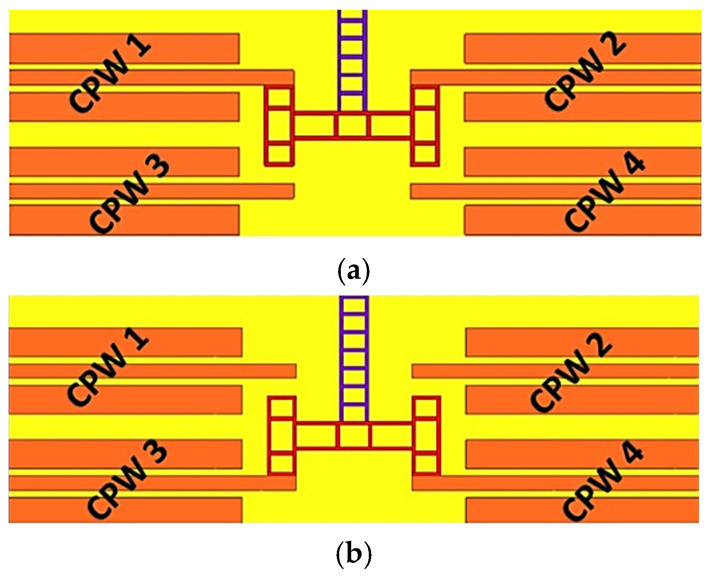
(**a**) STABLE STATE 1; (**b**) STABLE STATE 2.

In STABLE STATE 1, the lateral displacement of the H-clamp results in contact with CPW lines 1 and 2, as shown in Figure 2a. Transitioning to STABLE STATE 2 involves the lateral movement of the H-clamp to establish contact with CPW lines 3 and 4, as in Figure 2b. This movement is facilitated by the coordinated operation of the actuators. To revert the switch to its default configuration of STABLE STATE 1, the device employs its bimodal switching capability, which enables the switch to alternate between its two stable states efficiently. This bimodal switching plays a vital role in the device operation, allowing for controlled lateral movements of the H-clamp between distinct pairs of CPW lines, thereby determining the operational state of the switch. The S-parameters of a bistable lateral RF MEMS switch in both stable states are shown in Table 1.

### 2.3. Need for Structure Modification

The RF performance analysis of an H-clamp-based bistable RF MEMS switch was carried out in [34], and it was observed that the switch operates efficiently only up to 6 GHz. As wireless technologies advance, the operational frequencies increase, necessitating devices that can perform efficiently at higher frequencies. The H-clamp directly impacts the electromagnetic performance of the switch, influencing RF losses that are critical in high-frequency applications. Hence, structural change of the H-clamp solves the problem of achieving the stringent RF performance metrics required at high frequencies.

**Figure 3:**
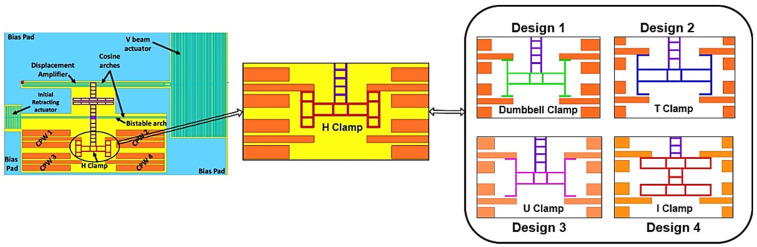
Structure modification of an H-clamp bistable RF MEMS switch with various lateral designs.

The H-clamp in a bistable switch is replaced with various reliable and mechanically stable lateral structures, such as dumbbell, T-shaped, U-shaped, and I-shaped clamps, as shown in Figure 3. Such lateral structures are designed and simulated in HFSS to analyze their RF performance. Among these structures, design 4 with an I-shaped clamp gives better RF performance when compared to the H-clamp structure.

Optimization of dimensional parameters of the proposed I-clamp bistable switch efficiently minimizes RF losses and ensures better signal transmission at higher frequencies. Conventionally, such optimization is performed in EM simulation tools, which is a complex, time-consuming process. Hence, a machine learning (ML) model was proposed to optimize the bistable switch to obtain better RF performance.

## 3. Optimization of an I-Clamp Bistable Lateral RF MEMS Switch Using a Machine Learning Model

Machine learning (ML) has transformed various domains to solve complex optimization problems. An ML model learns from previous data, constructs prediction models, and predicts the response whenever new data are processed. It employs various ML algorithms such as linear regression, logistic regression, support vector machines (SVMs), decision trees, random forest, gradient boosting machines (eXtreme Gradient Boost, LightGBM, CatBoost), k-nearest neighbors (KNN), naive Bayes, neural networks, and ensemble methods to identify relationships in data, enabling ML model development [34]. One major advantage of ML is that once a model is trained and achieves acceptable prediction accuracy, it can be used for future predictions without the need for retraining or additional adjustments. In [49,50,51,52,53], evolutionary algorithms, namely genetic algorithms and particle swarm optimization, were used to optimize MEMS switches by evaluating outputs and generating new search directions to find global maxima or minima. In contrast, ML focuses on predicting outputs based on the established input–output relationships, making it effective for future predictions without seeking global optima.

**Figure 4:**
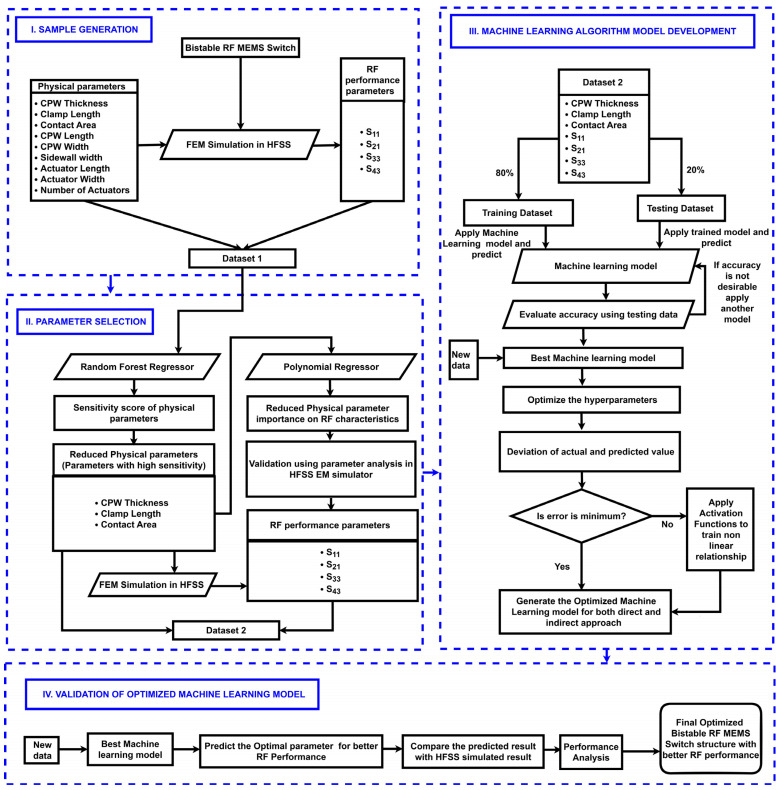
Proposed methodology for optimizing an I-clamp RF MEMS switch.

Implementing such ML algorithms to optimize the physical parameters of the proposed I-clamp-based RF MEMS switch significantly speeds up the design process. By predicting optimal configurations, ML provides a data-driven approach to designing a switch that is both efficient and innovative. The workflow of the proposed methodology for optimizing the I-clamp RF MEMS switch using an ML model is shown in Figure 4.

The proposed method consisted of the generation of data samples, design parameter selection for design space reduction, development of an ML model to optimize design parameters for achieving the required RF performance, and validation of the developed ML model.

### 3.1. Sample Generation

The proposed I-clamp RF MEMS switch was designed and simulated using a high-frequency structure simulator (HFSS), and the resulting datasets were extracted for ML-based optimization. These datasets were used to train ML models and evaluate them through testing and prediction. To generate a dataset, the nine physical parameters of the switch (Dataset 1), such as CPW thickness, length, and width, clamp length, contact area, sidewall width, actuator length and width, and number of actuators, were varied to a certain range of values, as shown in Table 2.

The S-parameter responses, including S_11_, S_21_, S_33_, and S_43_, were obtained from 1 to 10 GHz, generating 5000 data samples for Dataset 1.

### 3.2. Parameter Selection

The RF performance of the proposed I-clamp bistable RF MEMS switch is influenced by multiple physical design parameters. Exhaustively simulating all possible physical parameter combinations using full-wave EM solvers such as HFSS is computationally expensive. Although ML methods offer a faster alternative for design optimization, their effectiveness depends on identifying the most influential input parameters to reduce dimensionality and training overhead. To address this, a sensitivity score analysis was conducted to determine the influence of each physical parameter on the RF performance of the switch. Dataset 1, consisting of nine physical parameters, such as CPW thickness, length, and width, clamp length, contact area, sidewall width, actuator length, actuator width, and number of actuators, was considered as input and S-parameters as the output. A random forest regressor was employed due to its ability to model complex nonlinear relationships and its resistance to overfitting in high-dimensional data. Sensitivity scores were computed by varying one parameter at a time while keeping others constant, generating 2004 unique combinations of samples. The influence of each variable was evaluated based on its contribution to reducing prediction error across the ensemble of decision trees. This analysis reveals that CPW thickness, clamp length, and contact area have the highest impact on the RF performance. These parameters were selected for optimization, effectively reducing computational complexity and improving the efficiency of the design process.

The accuracy of the predicted output is directly proportional to the amount of data, as a larger number of data samples helps to develop a more precise predictive model. Hence, Dataset 2 was generated by varying the most influential parameters such as CPW thickness, clamp length, and contact area across a wide range of values. A large number of samples was obtained through full-wave EM simulations in HFSS to train the machine learning model effectively using diverse combinations of these critical parameters.

To evaluate the impact of the key physical parameters such as CPW thickness, clamp length, and contact area on each output S-parameter (S_11_, S_21_, S_33_, and S_43_), a polynomial regression model was employed. This method captures nonlinear relationships using a polynomial function of the form:

(1) $$y=\beta_{0}+\beta_{1}x+\beta_{2}x^{2}+\beta_{3}x^{3}+\cdots +\beta_{n}x^{n}+\epsilon$$

where y denotes the S-parameter, x represents the input physical parameters, β coefficients are learned during training, n is the polynomial degree, and ϵ is the error term. This approach enables a detailed understanding of how variations in physical dimensions influence specific RF responses, offering valuable insights for targeted optimization of the switch.

### 3.3. Machine Learning Model Development

To optimize the I-clamp bistable RF MEMS switch, Dataset 2 containing CPW thickness (CT), clamp length (CL), and contact area (CA) as the input physical parameters and S_11_, S_21_, S_33_, and S_43_ as the output RF parameters was split 80% for training and 20% for testing the ML models. Various ML models such as decision trees, random forests, artificial neural network (ANN), k-nearest neighbors (KNN), boosting algorithms such as adaptive boosting (ADABoost), categorical boosting (CATBoost), gradient boosting machine (GBM), eXtreme gradient boosting (XGBoost), and LightGBM were used to train Dataset 2. These algorithms were selected for their effectiveness in performing regression on nonlinear numerical datasets. Python 3 is the preferred programming language for implementing these algorithms due to its simplicity and the extensive availability of libraries that aid in data preprocessing, machine learning, and visualization.

**Figure 5:**
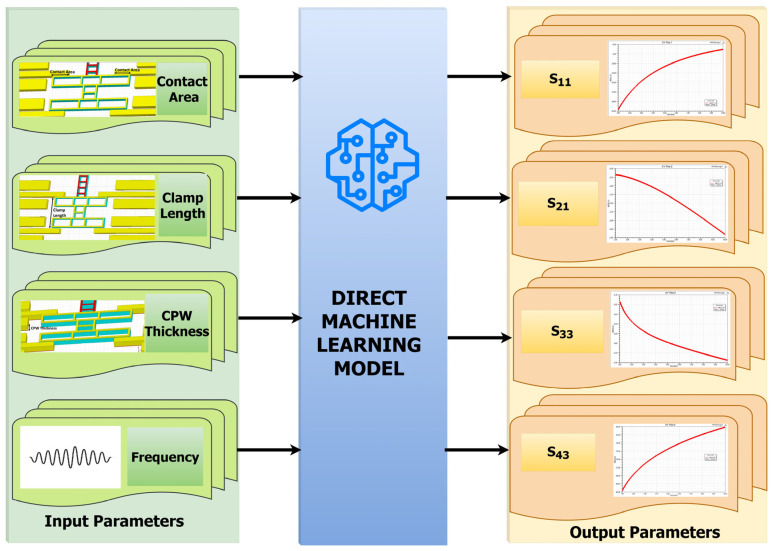
Direct ML model.

**Figure 6:**
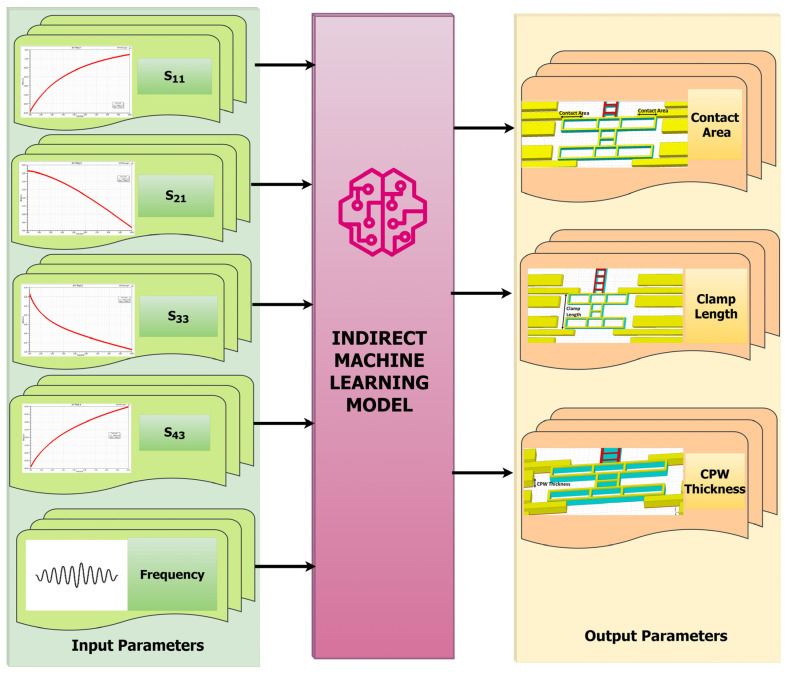
Indirect ML model.

When these ML models were trained and tested, the eXtreme gradient boosting (XGBoost) ML model provided high modeling accuracy, fast calculation speed, and strong generalization ability among various models. Since it has a high parameter extraction ability, it is beneficial to extract the subtle characteristics of a given switch. Hence, it was chosen for predicting the scattering parameters for any random combinations of dimensions at the desired frequency using the direct method, as in Figure 5, and for predicting the dimensions for any random combinations of scattering parameters using the indirect method, as in Figure 6, within the boundary limits defined by the training dataset.

**Figure 7:**
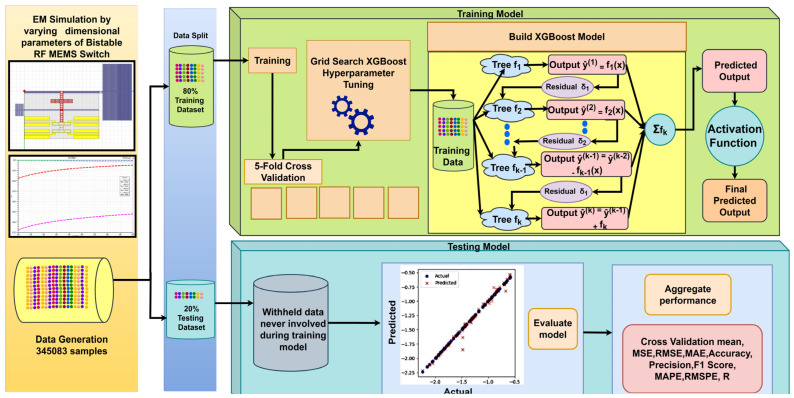
Methodology for developing an XGBoost-based ML model.

Figure 7 describes the methodology for developing and evaluating an XGBoost-based ML model; 345,083 samples generated by the HFSS EM simulator were split into 276,067 samples and 69,016 samples for training and testing the XGBoost ML model, respectively.

Prior to training the actual model, hyperparameter tuning was conducted using grid search, a method that exhaustively tests every combination of predefined sets of hyperparameter values such as column sampling by tree, minimum loss reduction (gamma), learning rate, maximum depth of a tree, minimum sum of instance weight needed in a child, number of trees or estimators, L_1_ regularization term on weights (alpha), L_2_ regularization term on weights (lambda), and subsample ratio of the training instances. Grid search systematically explores all possible combinations of hyperparameter values to identify the configuration that yields the best ML model performance.

To ensure model robustness and prevent overfitting, 5-fold cross-validation was employed. The training dataset was partitioned into five equal subsets, with each subset used once as a validation set while the remaining four were used for training. This process was repeated five times, ensuring each subset was used for validation exactly once. The performance metrics from all the folds were averaged to provide a reliable estimate of model generalization. This cross-validation strategy was combined with grid search to validate that the selected hyperparameters yielded consistent performance across different datasets. Table 3 gives the optimal values of hyperparameters for both the direct and indirect ML models using XGBoost.

Once the hyperparameters were determined, an XGBoost ML model was built by training a series of decision trees sequentially. Each tree *f*_1_, *f*_2_, …, *f_k_* tried to correct the errors such as residuals *δ*_1_, *δ*_2_, …, *δ_k_*_−1_ made by the previous tree in the series. The predicted output for the given samples was the sum of the predictions from all the trees. Mathematically, an XGBoost model can be represented as follows.

The XGBoost algorithm is complex and involves a series of linear combinations and an objective function optimized through gradient boosting. The training data D are represented as a set of tuples as in Equation (2), with the models in set Q consisting of $q_{1},\;q_{2},{\;q}_{3},\ldots \ldots \ldots ,\;q_{n}$.

(2) $$D=\{\left({CA}_{i},{CL}_{i},{CT}_{i},F_{i}\right),(S_{11i},S_{21i},S_{33i},S_{43i})\}$$

(3) $$Q=\{q_{1},q_{2},q_{3},\ldots \ldots \ldots ,\;q_{n}\}$$

Predictions are made through a linear combination of different models as in Equation (4).

(4) $$\left({\hat{S}}_{11i},{\hat{S}}_{21i},{\hat{S}}_{33i},{\hat{S}}_{43i}\right)=\sum_{t=1}^{m}q_{t}\left({CA}_{i},{CL}_{i},{CT}_{i},F_{i}\right)$$

Each instance i and model t produce an output ${\hat{S}}_{jit}$, with the objective function $O^{\left(t\right)}$ measuring the discrepancy between actual and predicted outputs using a differentiable convex loss function l.

(5) $$\begin{array}{cc} O^{\left(t\right)}= & \sum_{i=1}^{n}l\{\left(S_{11i},S_{21i},S_{33i},S_{43i}\right),\left({{\hat{S}}_{11i}}^{\left(t-1\right)},{{\hat{S}}_{21i}}^{\left(t-1\right)},{{\hat{S}}_{33i}}^{\left(t-1\right)},{{\hat{S}}_{43i}}^{\left(t-1\right)}\right)+\\ & q_{t}\left({CA}_{i},{CL}_{i},{CT}_{i},F_{i}\right)\}+\Omega \left(q_{t}\right) \end{array}$$

where $O^{\left(t\right)}$ is the objective function, l is a differentiable convex loss function used to measure the difference between the actual output, and predicted output, $\Omega (q_{t})$ is the regularization form. The introduction of the regularization term $\Omega (q_{t})$ mitigates overfitting by constraining model complexity, thereby promoting a simpler and more generalizable ML model. The objective function $O^{\left(t\right)}$ is expanded using Taylor series to approximate the loss, incorporating both the first- and second-order gradient statistics of the loss functions $g_{i}$ and $h_{i}$, respectively. These gradients are crucial in guiding the optimization process, allowing the algorithm to adjust its parameters intelligently and converge toward the minimum loss.

(6) $$f\left(a+h\right)=f\left(a\right)+f^{|}\left(a\right)h+\frac{1}{2}\;f^{\left|\right|}\left(a\right)h^{2}+f^{n}\left(a\right)\frac{h^{n}}{n!}$$

(7) $$\;a={{\hat{S}}_{11i}}^{\left(t-1\right)},{{\hat{S}}_{21i}}^{\left(t-1\right)},{{\hat{S}}_{33i}}^{\left(t-1\right)},{{\hat{S}}_{43i}}^{\left(t-1\right)}$$

(8) $$h=q_{t}\left({CA}_{i},{CL}_{i},{CT}_{i},F_{i}\right)$$

(9) $$\begin{array}{cc} O^{\left(t\right)}= & \sum_{i=1}^{n}l\left(S_{11i},S_{21i},S_{33i},S_{43i}\right),\left({{\hat{S}}_{11i}}^{\left(t-1\right)},{{\hat{S}}_{21i}}^{\left(t-1\right)},{{\hat{S}}_{33i}}^{\left(t-1\right)},{{\hat{S}}_{43i}}^{\left(t-1\right)}\right)+\\ & \left[\frac{\partial L\left(S_{11i},S_{21i},S_{33i},S_{43i}\right),\left({{\hat{S}}_{11i}}^{\left(t-1\right)},{{\hat{S}}_{21i}}^{\left(t-1\right)},{{\hat{S}}_{33i}}^{\left(t-1\right)},{{\hat{S}}_{43i}}^{\left(t-1\right)}\right)}{\partial \left({{\hat{S}}_{11i}}^{\left(t-1\right)},{{\hat{S}}_{21i}}^{\left(t-1\right)},{{\hat{S}}_{33i}}^{\left(t-1\right)},{{\hat{S}}_{43i}}^{\left(t-1\right)}\right)}\right]q_{t}\left({CA}_{i},{CL}_{i},{CT}_{i},F_{i}\right)+ \end{array}\;\frac{1}{2}\left(\frac{\partial L^{2}\left(S_{11i},S_{21i},S_{33i},S_{43i}\right),\left({{\hat{S}}_{11i}}^{\left(t-1\right)},{{\hat{S}}_{21i}}^{\left(t-1\right)},{{\hat{S}}_{33i}}^{\left(t-1\right)},{{\hat{S}}_{43i}}^{\left(t-1\right)}\right)}{\partial ({{\hat{S}}_{11i}}^{{\left(t-1\right)}^{2}},{{\hat{S}}_{21i}}^{{\left(t-1\right)}^{2}},{{\hat{S}}_{33i}}^{{\left(t-1\right)}^{2}},{{\hat{S}}_{43i}}^{{\left(t-1\right)}^{2}})}\right)q_{t}\left({CA}_{i},{CL}_{i},{CT}_{i},F_{i}\right)$$

(10) $$O^{\left(t\right)}=\sum_{i=1}^{n}\left\{C+g_{i}\;q_{t}\left({CA}_{i},{CL}_{i},{CT}_{i},F_{i}\right)\right\}+\frac{1}{2}h_{i}\;q_{t}{\left({CA}_{i},{CL}_{i},{CT}_{i},F_{i}\right)}^{2}+\Omega (q_{t})$$

where $g_{i}$ is the first-order gradient statistics of the loss function, $h_{i}$ is the second-order gradient statistics of the loss function. After introducing the training step, the complexity of the tree is defined as follows:

(11) $$q_{t}\left({CA}_{i},{CL}_{i},{CT}_{i},F_{i}\right)=w_{P(CA,CL,CT,F)},\;w\;\int R^{T},{R\;}^{d}\;\to \{1\ldots \ldots T\}$$

where *P* is the function assigned to each datapoint to leaf, *T* is the number of leaf nodes, and *w* is the vector of score for the leaves.

(12) $$\Omega \left(q_{t}\right)=\gamma T+\frac{1}{2}\;\lambda \sum_{j=1}^{k}{w_{j}}^{2}$$

(13) $$O^{\left(t\right)}=\sum_{i=1}^{n}\;[g_{i\;}w_{P({CA}_{i},{CL}_{i},{CT}_{i},F_{i})}+\frac{1}{2}h_{i}\;{w_{P({CA}_{i},{CL}_{i},{CT}_{i},F_{i})}}^{2}+\gamma T+\frac{1}{2}\;\lambda \sum_{j=1}^{k}{w_{j}}^{2}$$

The regularization term $\Omega \left(q_{t}\right)$ is composed of two parts: term γ which controls the number of leaves and term $\frac{1}{2}\;\lambda \sum_{j=1}^{k}{w_{j}}^{2}$, which controls the scores on the leaves. The final objective $O^{\left(t\right)}$ then combines the predictions weighted by the gradient statistics with the regularization term to arrive at a value that the algorithm seeks to minimize the loss.

The capacitance C of the switch when the I-clamp and CPW lines are decoupled are as follows:

(14) $$C=\frac{\epsilon_{r}\epsilon_{o}A}{d}$$

where $\epsilon_{r}$ and $\epsilon_{o}$ are the permittivities of the dielectric and the vacuum, respectively, A represents the area, and d is the gap distance. The capacitance exhibits a hyperbolic dependency on d, significantly influencing the insertion loss when the switch is in the open position. This relationship highlights the nonlinear characteristics of the RF performance of the switch as dimensions are varied slightly. The impedance Z of the switch when the I-clamp and CPW lines come in contact with each other is as follows:

(15) $$Z\approx \;\frac{L}{W\cdot T\cdot \sigma }$$

where L is the length, W is the width, T is the thickness of the conductive path, and σ is the conductivity of the material. Impedance plays a crucial role in minimizing reflections and optimizing transmission. Hence, the S_11_ and S_21_ parameters’ dependence on the physical dimensions of the switch demonstrates nonlinear characteristics, especially notable in high-frequency operations where skin and proximity effects are significant. Such intricate and nonlinear relationships between the physical dimensions of RF MEMS switches and their associated S-parameters make the prediction of physical parameters from the RF performance more sensitive. Since the inverse modeling of RF MEMS switches with nonlinear characteristics is quite complex to achieve more accuracy, activation functions introduce nonlinearity to the XGBoost ML model, enabling it to learn complex patterns beyond simple linear decision boundaries. They also play a critical role in the model by allowing gradients to be propagated through the decision trees, facilitating effective learning and updates to the regularization term as in Equation (16):

(16) $$\left(\hat{\lambda },\hat{\gamma },\hat{T}\right)=\arg\min F\left(\lambda_{i},\;\gamma_{j},T_{k}\right)$$

Thus, Equation (13) with the activation function becomes the following:

(17) $$O^{\left(t\right)}=\sum_{i=1}^{n}\;[F(g_{i\;}w_{P\left({CA}_{i},{CL}_{i},{CT}_{i},F_{i}\right)})+\frac{1}{2}h_{i}\;{{F(w}_{P({CA}_{i},{CL}_{i},{CT}_{i},F_{i})}}^{2})+\hat{\gamma }\hat{T}+\frac{1}{2}\;\hat{\lambda }\sum_{j=1}^{k}F({w_{j}}^{2})$$

Various activation functions were applied to the sum of the trained outputs to obtain the final predicted output with more accuracy. The activation function that enhanced the prediction accuracy of both the direct and indirect modeling of the proposed XGBoost model was further used for the optimization of the I-clamp RF MEMS switch. While the developed machine learning model demonstrates strong predictive performance, the inclusion of physical knowledge can further enhance interpretability and generalization. The following section discusses how physics-informed and hybrid approaches can complement the data-driven methodology.

### 3.4. Augmenting Data-Driven Models with Physical Constraints

Physics-informed machine learning (PIML) integrates known physical relationships into the training process to ensure the model adheres to established device behavior. For MEMS devices, this means incorporating expressions such as capacitance versus gap C(x)∝1/x and impedance versus geometry Z∝√(L/WT) directly into the loss function. This constrains the model to produce outputs that satisfy physical laws throughout training, not just fit the data. Enhanced versions, such as gradient-informed physics-informed neural networks (PINNs), also include derivative information—for example, penalizing violations in dC/dx or dZ/dx to improve performance in regions with steep physical changes, such as during contact in MEMS switches. Hybrid (grey-box) learning methods combine fast physics-based estimates with machine learning to capture effects not covered by simplified models. A typical workflow computes a baseline prediction ${\hat{S}}_{\mathrm{phys}}$ using reduced-order models or closed-form approximations. A learning algorithm, such as XGBoost, is then trained on the residual error $\Delta S=S_{\mathrm{HFSS}}-{\hat{S}}_{\mathrm{phys}}$, where $S_{\mathrm{HFSS}}$ is the output from a high-frequency structure (HFSS). The final prediction is constructed as follows:

(18) $$S_{\mathrm{pred}}={\hat{S}}_{\mathrm{phys}}+\Delta S$$

This approach blends first-principles and data-driven modeling, improving extrapolation to new geometries and accounting for parasitic and layout-level non-idealities that analytical models might miss. Such methods not only enhance physical consistency, but also reduce overfitting and improve robustness—addressing the need for models that go beyond purely statistical “black-box” approaches. Building on these physics-aware strategies, we can also explore data-efficient sampling methods to further reduce our simulation burden.

### 3.5. Active Learning and Surrogate Modeling

Active learning and surrogate modeling naturally extend hybrid frameworks by focusing computational effort where it yields the greatest insight. To reduce reliance on exhaustive HFSS simulations, one can adopt an active learning-driven surrogate workflow. Starting from a small set of HFSS runs, a fast surrogate—such as a Gaussian process or neural network—predicts S-parameters across the design space. The surrogate’s uncertainty estimates then guide the selection of the next HFSS simulation points, concentrating efforts where they add the most value and drastically reducing the total number of runs. Surrogate-based optimization methods—such as expected improvement infill criteria—have long been used in electromagnetic design to approximate expensive black-box functions and drive sequential sampling. Recent studies have shown that active learning can achieve relative errors below 0.1% with fewer than fifty full-wave simulations, demonstrating its suitability for RF MEMS switch optimization. Integrating this strategy into our workflow would enable efficient dataset expansion by steering HFSS runs toward the most informative geometries. With this efficient data-generation scheme in place, we now turn to validate the predictive performance of our models.

### 3.6. Validation of the Machine Learning Model

Once both the direct and indirect ML models had been trained, they were tested using the withheld testing dataset that had never been involved in the training process. The performance of the model was evaluated by comparing the actual values from HFSS simulations with the predicted values from the ML model. A scatter plot comparing the actual values versus predicted values was used to evaluate prediction accuracy, with data closely aligned along the diagonal line indicating the ideal prediction accuracy.

The performance of various regression models is evaluated quantitatively using metrics such as mean square error (MSE), root mean square error (RMSE), mean absolute error (MAE), accuracy, training time, mean absolute percentage error (MAPE), root mean square percentage error (RMSPE), and the correlation coefficient (R). Each metric provides distinct insights into the predictive capabilities of the ML models. MSE and RMSE were used to evaluate the average magnitude of the ML model errors, with lower values indicating closer adherence of predictions to actual values.

MAE aided in quantifying the average prediction error, offering a direct measure of the average error magnitude. MAPE and RMSPE were used to assess errors in a relative sense, which is crucial for datasets with varying ranges. R was calculated to gauge the strength of the linear relationship between the predicted and actual values. Accuracy shows the proportion of predictions falling within a predefined acceptable range of the actual values. Training time was monitored to evaluate the computational efficiency of the model, an important factor in practical applications.

**Figure 8:**
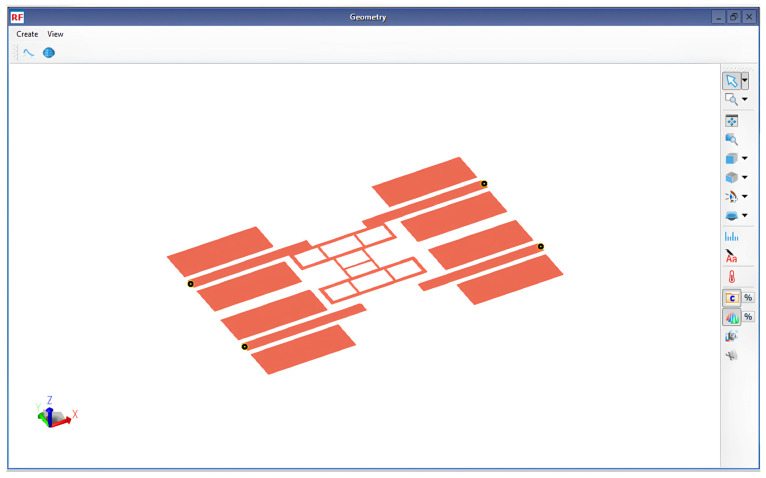
Layout of the proposed I-clamp RF MEMS switch in RFPro of ADS.

To validate the accuracy of the proposed I-clamp RF MEMS switch, post-layout parasitic extraction-based modeling was conducted to observe the performance closer to the fabricated prototype. The layout of the I-clamp switch was simulated in the RFPro platform of Advanced Design System (ADS), as shown in Figure 8.

**Figure 9:**
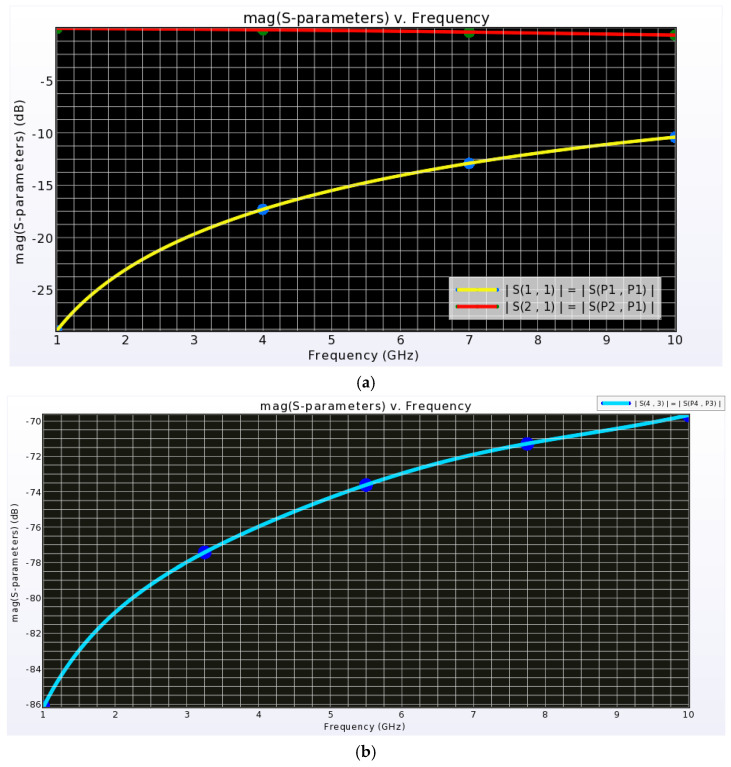
Post-layout extracted S-parameters of the I-clamp RF MEMS switch. (**a**) Return loss (S_11_) and insertion loss (S_21_). (**b**) Isolation loss (S_43_).

The simulation was carried out at an ambient temperature of 25 °C, with the operating frequency sweep from 1 GHz to 10 GHz. This setup was used to extract return loss, insertion loss, and isolation of the proposed switch, as shown in Figure 9.

**Figure 10:**
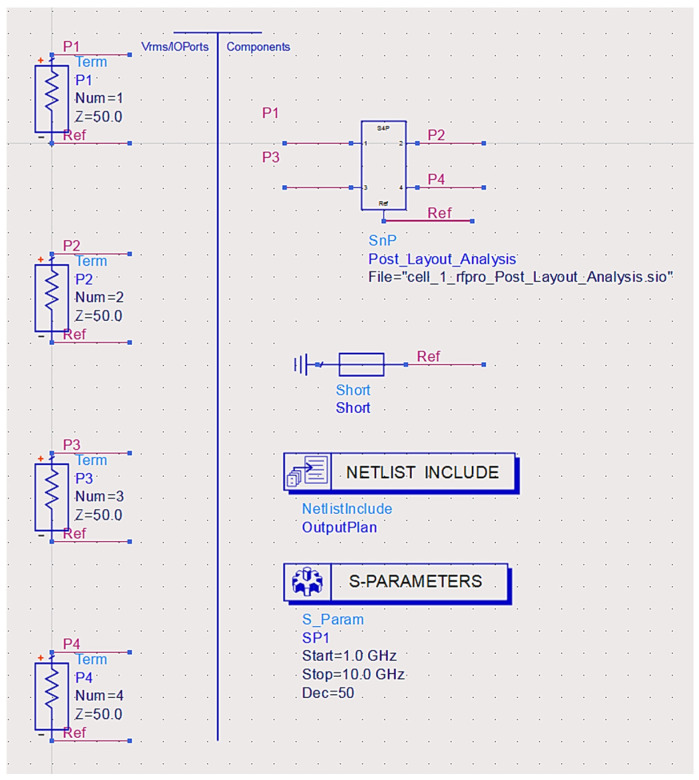
Post-layout extraction simulation setup with a netlist-based test bench.

A test bench was created for the post-layout I-clamp RF MEMS switch in ADS to simulate the parasitic-extracted netlist using boundary conditions and excitation ports, as shown in Figure 10, to evaluate the S-parameter performance.

**Figure 11:**
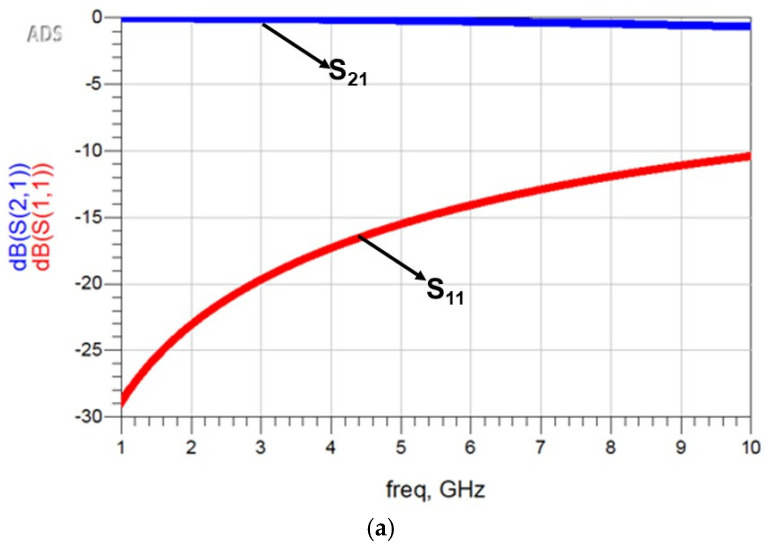

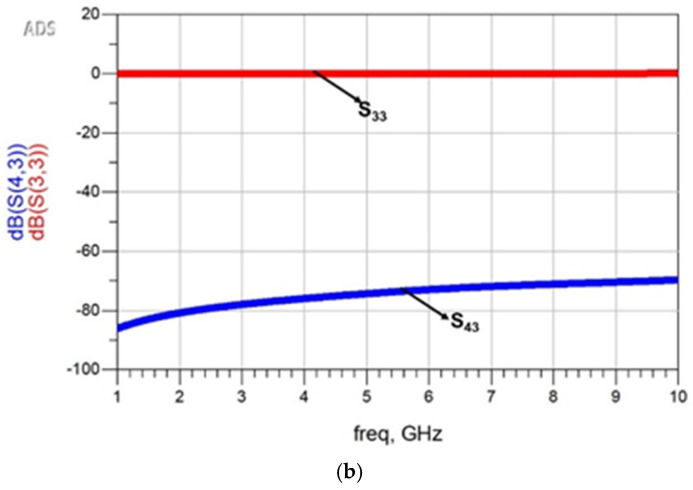
S-parameters of the proposed I-clamp RF MEMS switch from the ADS netlist-based test bench simulation. (**a**) S_11_ and S_21_. (**b**) S_33_ and S_43_.

The test bench simulation results of the parasitic-extracted netlist are shown in Figure 11. These results closely align with the full-wave EM simulation.

## 4. Results and Discussion

**Figure 12:**
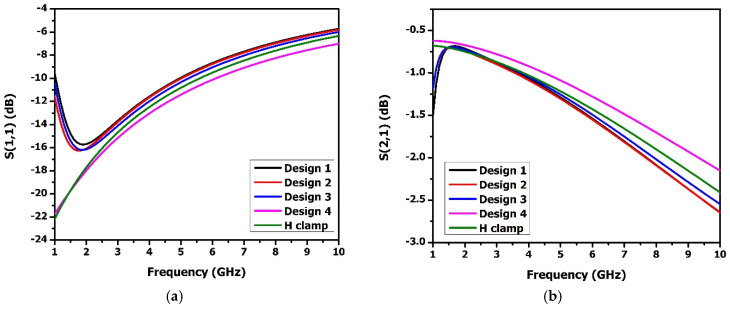

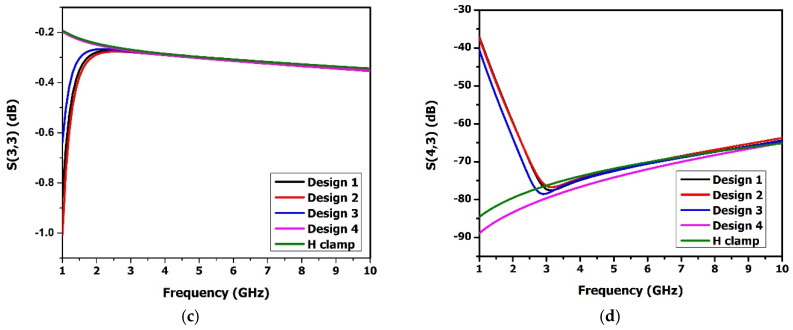
S-parameter comparison of various lateral designs of the bistable RF MEMS switch. (**a**) S_11_, (**b**) S_21_, (**c**) S_33_, (**d**) S_43_.

The electrothermally actuated bistable lateral RF MEMS switch structure used for low-frequency wireless applications was modified to improve its RF performance at a higher frequency. Structural modifications to the H-clamp enhanced its electromagnetic performance, reducing RF losses essential for high-frequency applications, thus meeting stringent RF performance requirements. Thus, the H-clamp structure of the switch was replaced by various lateral switch structures, and it was observed that the I-clamp structure showed better insertion loss and isolation. As shown in Figure 12, the I-clamp (design 4) bistable RF MEMS switch’s S_11_ was −10.5 dB, S_21_—−1.5 dB, S_33_—−0.3 dB, and S_43_—−74 dB at 7 GHz.

To further improve the RF performance of the proposed I-clamp bistable RF MEMS switch, optimization of the design parameters was carried out. The optimization of the I-clamp bistable switch through ML models streamlined the traditionally complex and time-consuming EM simulation process, minimizing RF losses and enhancing signal transmission at higher frequencies. To train the ML model, Dataset 1 consisting of 2004 data samples with RF performance of the switch was obtained from the HFSS EM simulator by varying the physical parameters such as CPW thickness (CT), length (L), and width (W), clamp length (CL), contact area (CA), sidewall width (SW), actuator length (AL) and width (AW), and number of actuators (AN), as shown in Table 4.

**Figure 13:**
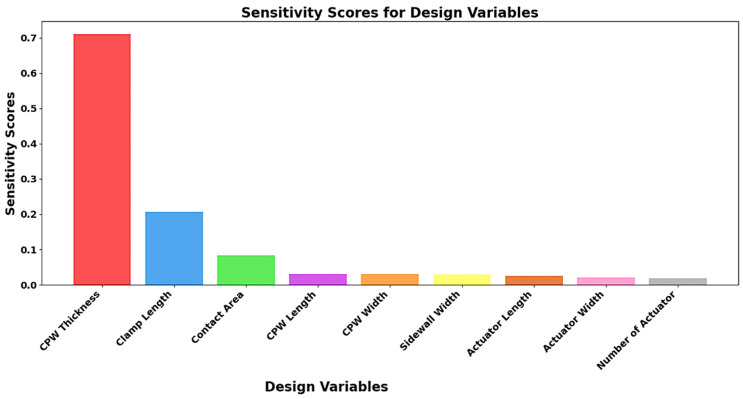
Sensitivity scores of the bistable RF MEMS switch design variables.

The design space for optimizing the proposed RF MEMS switch was reduced using sensitivity score analysis, which identified the parameters that significantly impacted RF performance. Dataset 1, containing physical parameters as inputs and S-parameters as outputs, was analyzed with a random forest regressor. This model streamlined the optimization process by identifying less significant design variables that could be excluded with minimal impact on RF performance, as shown in Figure 13.

From Figure 13, it is observed that CPW thickness, clamp length, and contact area had high sensitivity scores, indicating a significant impact on the performance of the I-clamp RF MEMS switch. Therefore, these input parameters were selected for the optimization of the switch. Dataset 2, with a large number of samples, was generated by varying these selected input parameters, as shown in Table 5, for training both direct and indirect ML models.

**Figure 14:**
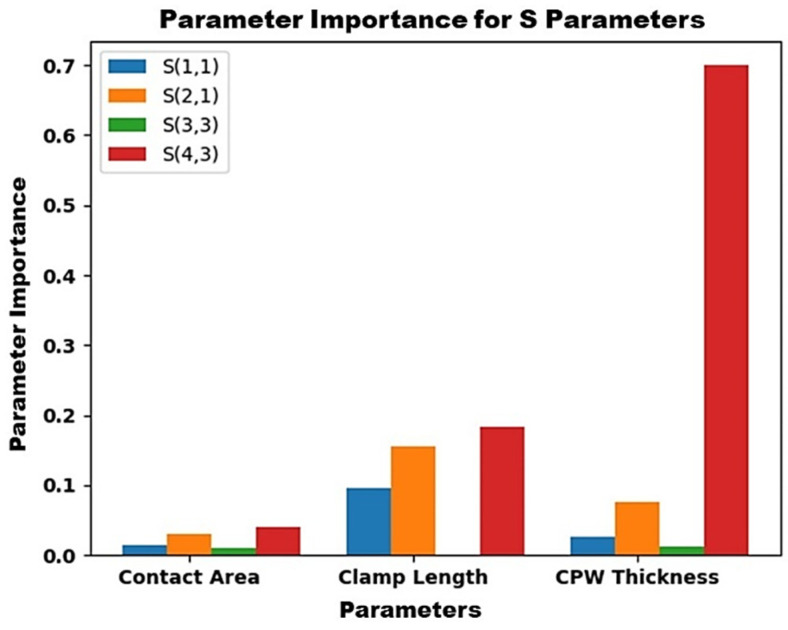
Bistable RF MEMS switch parameter importance for the S-parameters.

The influence of each input physical parameter on the individual output S-parameters was analyzed using a polynomial regressor, and it was observed that isolation was greatly influenced by input parameters, as shown in Figure 14.

Dataset 2 with 345,083 samples was split 80% for training and 20% for testing. The direct and indirect ML models were trained using various machine learning algorithms such as decision tree, random forest, ANN, KNN, ADABoost, CATBoost, GBM, XGBoost, and LightGBM. The ML algorithm that provided the better performance was selected for optimization. Table 6 compares the performance metrics of the various ML algorithms for both direct and indirect methods.

Table 6 shows that the XGBoost-based ML model performed efficiently compared to the other algorithms since it consistently demonstrated superior performance metrics, such as lower MSE, RMSE, MAE, MAPE, RMSPE values, and higher accuracy percentages and R^2^ values for both direct and indirect methods. This advantage was due to the ability of XGBoost to handle missing data, prevent overfitting with regularization, and utilize parallel processing for faster computation.

The prediction accuracy of the indirect model, which aimed to predict dimensions from S-parameters using an ML model, was lower than that of the direct model due to the complexity and inherent challenges in capturing the inverse relationship. Since it was highly nonlinear and sensitive, this complexity increased the potential for errors and inaccuracies, leading to lower accuracy in predictions. To address this issue, we proposed a model in which activation functions were used in conjunction with the XGBoost model. Various activation functions introduced nonlinearity into the model, enabling it to capture more complex patterns and relationships within the data.

Selecting an appropriate activation function is key for effective training and performance in ML model-based regressor problems. In regression models, activation functions must be nonlinear to capture complex patterns, differentiable for gradient-based optimization, zero-centered for balanced weight updates, and resistant to vanishing and exploding gradients to maintain effective learning and numerical stability. The trade-off between these characteristics is observed for different activation functions to choose the efficient activation function, which has such advantages as smooth gradients, efficient computation, and robustness against common training pitfalls. Table 7 provides a comparison of activation functions and evaluates their performance based on these key characteristics, which are essential in determining the effectiveness of each activation function in the regression problem.

Based on the comparative analysis of activation function characteristics, as shown in Table 7, functions lacking key features such as differentiability, zero-centered output, or resistance to vanishing and exploding gradients were excluded from further optimization. These included Binary Step, Sigmoid, Softmax, Hard Sigmoid, Squared ReLU, and Log Softplus Error. This analysis focused on a subset of activation functions demonstrating strong theoretical and empirical suitability for regression modeling in RF MEMS switch optimization. The proposed XGBoost ML model was trained with different activation functions, and MSE values were obtained, as shown in Table 8.

**Figure 15:**
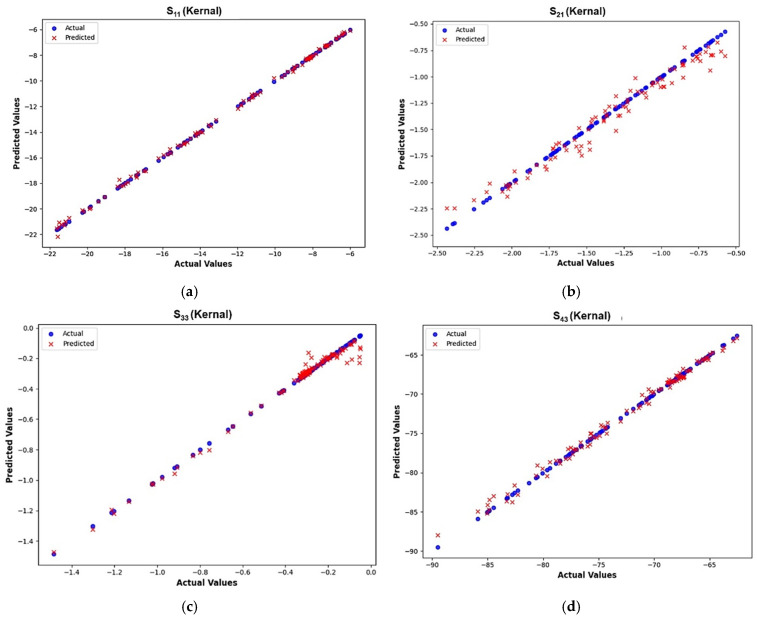
Actual vs. predicted values obtained from the kernel-based XGBoost direct ML model. (**a**) S_11_. (**b**) S_21_. (**c**) S_33_. (**d**) S_43_.

**Figure 16:**
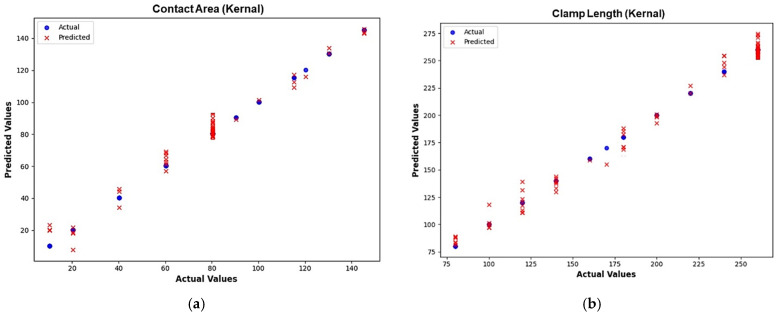

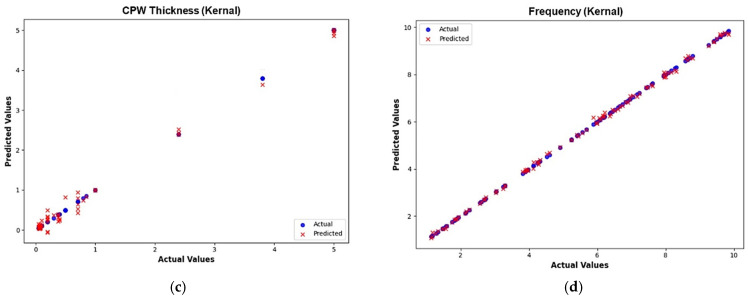
Actual vs. predicted values obtained from the kernel-based XGBoost direct ML model. (**a**) Contact area. (**b**) Clamp length. (**c**) CPW thickness. (**d**) Frequency.

In Table 8, the integration of the kernel function with the XGBoost ML model gives a minimum prediction error of 0.127, and hence it was chosen for optimizing the I-clamp bistable RF MEMS switch. This ensured that the kernel activation function optimally improved the XGBoost model performance, effectively capturing the complex dependencies and reducing prediction errors in the indirect model. The proposed kernel-based XGBoost ML regressor model efficiently handled the intricate and nonlinear relationship between S-parameters and physical dimensions, enhancing its ability to make more accurate predictions despite the complexity of the indirect model. The XGBoost-based ML model with the kernel activation function was selected for prediction in both direct and indirect ML models. It was found that XGBoost converged well with the HFSS-simulated values, as shown in Figure 15 and Figure 16.

**Figure 17:**
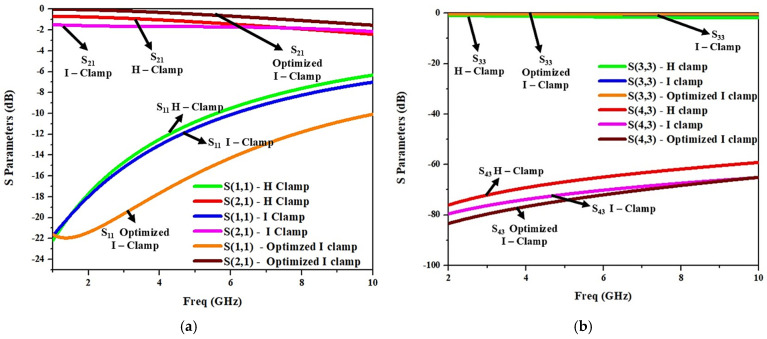
S-parameter results of the optimized bistable RF MEMS switch. (**a**) S_11_ and S_21._ (**b**) S_33_ and S_43_.

The proposed kernel-based XGBoost machine learning model achieved rapid prediction of RF performance metrics within 8 min, even for large datasets, whereas conventional HFSS simulations required multiple iterations, each taking approximately 65 min on a system with 8 GB RAM and a 2.90 GHz Intel Core processor. This approach resulted in an 87.7% reduction in design time for the I-clamp bistable RF MEMS switch compared to traditional full-wave EM simulations. The proposed I-clamp bistable RF MEMS switch that had better RF performance was predicted using the proposed kernel-based XGBoost ML model, and it was predicted that it performed well with the CPW thickness of 0.5 µm, contact area of 145 µm, and clamp length of 260 µm. It was observed that the proposed structure ensured better RF performance, as shown in Figure 17. Table 9 gives a state-of-the-art comparison of bistable RF MEMS switches with the proposed switch.

From Table 9, it is observed that the RF performance of the proposed I-clamp switch was comparable to certain existing designs, demonstrating significantly higher isolation (−70 dB) and improved return loss (−11 dB) at 10 GHz. Unlike most existing studies, our work utilized artificial neural network (ANN) and machine learning (ML) models to predict the optimal dimensions of the switch by reducing design time, as highlighted in Table 9. In [34], although the ANN model achieved 99.5% accuracy in predicting one physical parameter, it could not handle the simultaneous prediction of multiple design parameters. Our proposed work leverages an XGBoost ML-based optimization strategy with a kernel activation function, which reduced the design time by 87.7% and enabled accurate prediction of optimal multidimensional structural parameters at the same time. To validate the simulation accuracy, post-layout parasitic extraction-based modeling was performed. Table 10 shows the comparison of EM simulation and post-layout S-parameters at 10 GHz, confirming the robustness of the proposed design through better RF performance.

The fabrication and experimental characterization of the proposed I-clamp bistable RF MEMS switch will be carried out to validate the simulation-based findings in the future. The ML models developed in this work and trained on HFSS simulation data can be extended to incorporate experimental S-parameter measurements. This will enable the refinement of the model by capturing real-world variations arising from fabrication tolerances, material imperfections, and environmental influences. With sufficient measured data, the ML model can be retrained to enhance its predictive accuracy, thereby supporting more reliable and practically optimized switch designs.

## 5. Conclusions

This paper proposes an I-clamp bistable RF MEMS switch that significantly enhances RF performance in wireless communication systems, particularly by reducing insertion loss and increasing isolation. Optimization of the I-clamp structure by identifying the critical physical parameters that affect switch performance enabled us to efficiently explore a vast design space and identify optimal configurations that enhance RF efficiency. This work addresses the limitations of conventional optimization methods, which are often time-consuming and computationally intensive. The XGBoost ML model with the kernel activation function proved to be highly effective in predicting and optimizing these parameters. The ability of the ML model with an activation function to learn the complex, nonlinear relationships between physical dimensions and RF characteristics was instrumental in achieving superior performance metrics. The results from HFSS simulations and XGBoost predictions converged well with errors less than 0.5, proving the efficiency of the developed ML optimization approach. The optimized I-clamp bistable lateral RF MEMS switch showed better RF performance up to 10 GHz, making it reliable for high-frequency wireless communication. This ML approach saves time and computational resources while achieving better RF performance, leading to more efficient and reliable high-frequency applications.

## Figures and Tables

**Table 1:** S-parameters of a bistable lateral RF MEMS switch in both stable states.

Stable State	Return Loss	Insertion Loss	Isolation Loss
1	S_11_	S_21_	S_43_
2	S_33_	S_43_	S_21_

**Table 2:** Boundary conditions of the physical parameters for dataset generation.

Physical Parameter	Lower Limit	Upper Limit
CPW thickness	0.01 µm	10 µm
CPW length	652 µm	850 µm
CPW width	30 µm	50 µm
Clamp length	80 µm	270 µm
Contact area	80 µm	150 µm
Sidewall width	0.1 µm	0.5 µm
Actuator length	500 µm	1500 µm
Actuator width	1 µm	20 µm
No. of actuators	10	100

**Table 3:** Optimal hyperparameter values for the XGBoost models using direct and indirect methods.

Hyperparameters	Direct Method	Indirect Method
	Optimal Values	
colsample bytree	0.871	0.818
gamma	0.140	0.725
learning rate	0.178	0.291
max depth	5	7
min child weight	4.165	2.376
n estimators	380	154
reg alpha	0.080	0.771
reg lambda	0.794	0.765
subsample	0.777	0.961

**Table 4:** Sample Dataset 1 containing physical parameters and the corresponding RF performance.

Physical Parameters									RF Performance				
CT (µm)	L (µm)	W (µm)	CL (µm)	CA (µm)	SW (µm)	AL (µm)	AW (µm)	AN	F (GHz)	S_11_ (dB)	S_21_ (dB)	S_33_ (dB)	S_43_ (dB)
1	800	40	260	80	0.1	500	1	10	10	−5.2	−4	−0.4	−23
0.5	750	40	152	145	0.1	1500	20	100	4	−16	−1	−0.3	−80
8	800	35	98	100	0.25	1450	10	80	2	−20	−0.6	−0.2	−85

**Table 5:** Input and output parameters to train the direct and indirect ML models.

Direct Method							
Input Parameters				Output Parameters			
Contact Area (µm)	Clamp Length (µm)	CPW Thickness (µm)	Freq (GHz)	S_11_ (dB)	S_21_ (dB)	S_33_ (dB)	S_43_ (dB)
80	80	5	1.18	−20.47	−0.548	−0.051	−81.03
80	218	0.2	6.87	−9.150	−1.407	−0.648	−70.39
100	220	0.38	9.87	−7.23	−2.090	−0.441	−64.02
20	220	0.08	5.9	−11.65	−1.280	−1.130	−71.54
75	130	0.5	3.98	−12.26	−0.928	−0.283	−71.64
**Indirect Method**							
**Input Parameters**					**Output Parameters**		
**S_11_** **(dB)**	**S_21_** **(dB)**	**S_33_** **(dB)**	**S_43_** **(dB)**	**Freq** **(GHz)**	**Contact Area** **(µm)**	**Clamp Length** **(µm)**	**CPW Thickness** **(µm)**
−6.50	−2.303	−0.339	−68.24	10	10	220	0.5
−14.02	−0.888	−0.103	−75.41	3.9	145	219	0.45
−20.3	−0.601	−0.207	−80.67	1.2	40	120	0.55
−8.050	−1.674	−0.233	−65.95	7.2	80	100	0.71
−12.47	−0.995	−0.159	−76.32	4.31	77	222	1.05

**Table 6:** Performance metrics of the various ML algorithms for the direct and indirect methods.

Performance Metrics	Direct Method								
	Decision Tree	Random Forest	ANN	KNN	XGB	ADA	CAT	GBM	LightGBM
MSE	0.897	1.123	1.567	1.789	0.187	3.189	0.246	0.255	0.255
RMSE	0.532	0.678	0.894	0.923	0.295	1.448	0.139	0.150	0.1522
MAE	0.621	0.734	0.856	0.912	0.071	1.055	0.041	0.045	0.0511
R^2^	0.899	0.923	0.945	0.967	0.996	0.773	0.997	0.997	0.997
Accuracy (%)	97.65	98.45	98.78	98.90	99.83	48.266	96.55	96.121	95.66
MAPE	5.678	6.789	7.123	7.456	3.070	38.313	2.118	2.584	2.944
RMSPE	45.678	56.789	67.123	78.456	30.403	113.609	13.572	26.465	37.423
**Performance Metrics**	**Indirect Method**								
	**Decision Tree**	**Random Forest**	**ANN**	**KNN**	**XGB**	**ADA**	**CAT**	**GBM**	**LightGBM**
MSE	10.567	12.789	14.123	16.789	5.424	1019	138.943	158.206	76.569
RMSE	11.678	14.345	16.789	18.123	9.768	276.11	115.697	10.782	6.200
MAE	9.123	10.567	12.345	13.678	4.719	19.079	7.714	3.485	4.14
R^2^	0.745	0.812	0.834	0.856	0.863	0.415	0.853	0.892	0.889
Accuracy (%)	88.45	90.78	92.12	94.56	94.941	25.083	41.15	44.249	43.622
MAPE	36.789	40.123	43.456	45.789	33.59	68.797	33.036	27.791	26.807
RMSPE	178.789	190.123	200.456	210.789	228.21	157.704	136.012	128.029	121.85

**Table 7:** Comparative evaluation of activation functions based on the regression model performance characteristics.

Activation Function	Nonlinear	Differentiable	Zero-Centered	Vanishing Gradient-Resistant	Exploding Gradient-Resistant
Binary Step	✓	✗	✗	✗	✗
Sigmoid	✓	✓	✗	✗	✗
Tanh	✓	✓	✓	✓	✗
Arctan	✓	✓	✓	✓	✗
ReLU	✓	✓	✗	✓	✗
Parametric ReLU	✓	✓	✗	✓	✗
ELU	✓	✓	✓	✓	✗
Softmax	✓	✓	✗	✗	✗
Leaky ReLU	✓	✓	✗	✓	✗
Randomized ReLU	✓	✓	✗	✓	✗
SELU	✓	✓	✓	✓	✓
Linear Unit	✗	✓	✓	✓	✓
Swish	✓	✓	✓	✓	✓
Hard Sigmoid	✓	✓	✗	✗	✗
GELU	✓	✓	✓	✓	✓
Mish	✓	✓	✓	✓	✓
SiLU	✓	✓	✓	✓	✓
ReLU6	✓	✓	✗	✓	✓
Hard Swish	✓	✓	✓	✓	✓
Maxout	✓	✓	✓	✓	✓
Shifted Softplus	✓	✓	✓	✓	✓
Concatenated ReLU	✓	✓	✗	✓	✓
Squared ReLU	✓	✓	✗	✗	✗
Tanh Exponential	✓	✓	✓	✓	✗
Kernel	✓	✓	✓	✓	✓
Polynomial Kernel	✓	✓	✓	✓	✓
SReLU	✓	✓	✓	✓	✓
Log Softplus Error	✓	✓	✗	✗	✗
PELU	✓	✓	✓	✓	✓
Elish	✓	✓	✓	✓	✓
SerReLU	✓	✓	✓	✓	✓

**Table 8:** Mean square error of the various activation functions.

Activation Function	MSE
tanh	814.13
arctan	1.59 × 10^24^
relu	1367.04
parametric relu	1369
exponential linear unit	10,285,604
softplus	1936.77
leaky_relu	1369
randomized relu	1375
scaled exponential linear_unit	10,285,604.19
swish	1345.36
gaussian error linear unit	121
mish	1258
selu	1375
sigmoid linear unit	1365
relu6	1363
hard swish	1345
maxout	1367
shifted softplus	2064
concatenated relu	1363
tanh exp	130
kernel	0.12
polynomial kernel	316
srelu	42,278,321
pelu	761,599,066
elish	1361
serrelu	1357
siren	1360

**Table 9:** State-of-the-art comparison of bistable RF MEMS switches with the proposed I-clamp design.

Ref.	Actuation Type	Structure	Insertion Loss (dB)	Isolation (dB)	Return Loss (dB)	Frequency (GHz)	Design Tools Used
Zhang et al. [54]	Electromagnetic	Cantilever beam	−0.11	−43	-	3	ANSYS
Sun et al. [55]	Electrostatic	Two cantilevers	− 1.9	− 33.18	-	6	HFSS
Daneshmand et al. [56]	Electrothermal	Bridge	−0.8	−20	−20	20	COMSOL
Pirmoradi et al. [32]	Electrothermal	Slider	−1	−20	−10	140	HFSS and COMSOL
Naito et al. [57]	Electrostatic	Movable electrodes	−0.5	−30	-	5	ADS
^†^ Percy et al. [34]	Electrothermal	H-clamp	−1.7	−65	−10	6	HFSS and ANN model
^†^ This work	Electrothermal	I clamp	−0.8	−70	−11	10	HFSS and kernel-based XGBoost model

Note: ^†^ these works utilized ML models to reduce the design time of the RF MEMS switch.

**Table 10:** EM simulation and post-layout S-parameter comparison at 10 GHz for the proposed I-clamp RF MEMS switch.

Analysis	Insertion Loss (dB)	Isolation (dB)	Return Loss (dB)
EM simulation	−0.8	−70	−11
Post-layout	−0.65	−70	−10.5

## Data Availability

The original contributions presented in this study are included in the article. Further inquiries can be directed to the corresponding author.
